# *tkt1*, located on a novel pathogenicity island, is prevalent in avian and human extraintestinal pathogenic *Escherichia coli*

**DOI:** 10.1186/1471-2180-12-51

**Published:** 2012-04-03

**Authors:** Ganwu Li, Subhashinie Kariyawasam, Kelly A Tivendale, Yvonne Wannemuehler, Christa Ewers, Lothar H Wieler, Catherine M Logue, Lisa K Nolan

**Affiliations:** 1 Department of Veterinary Microbiology and Preventive Medicine, College of Veterinary medicine, Iowa State University, 1802 University Blvd., VMRI 2, Ames, Iowa 50011, USA; 2 Department of Veterinary and Biomedical Sciences, Pennsylvania State University, University Park, PA 16802, USA; 3 Faculty of Veterinary Science, The University of Melbourne, Parkville, Melbourne, Victoria 3010, Australia; 4 Institute of Microbiology and Epizootics, Free University Berlin, Philippstraße 13, D-10115 Berlin, Germany

## Abstract

**Background:**

Extraintestinal pathogenic *Escherichia coli *are important pathogens of human and animal hosts. Some human and avian extraintestinal pathogenic *E. coli *are indistinguishable on the basis of diseases caused, multilocus sequence and phylogenetic typing, carriage of large virulence plasmids and traits known to be associated with extraintestinal pathogenic *E. coli *virulence.

**Results:**

The gene *tkt1 *identified by a previous signature-tagged transposon mutagenesis study, was found on a 16-kb genomic island of avian pathogenic *Escherichia coli *(APEC) O1, the first pathogenic *Escherichia coli *strain whose genome has been completely sequenced. *tkt1 *was present in 39.6% (38/96) of pathogenic *Escherichia coli *strains, while only 6.25% (3/48) of *E. coli *from the feces of apparently healthy chickens was positive. Further, *tkt1 *was predominantly present in extraintestinal pathogenic *E. coli *belonging to the B2 phylogenetic group, as compared to extraintestinal pathogenic *E. coli *of other phylogenetic groups. The *tkt1*-containing genomic island is inserted between the *metE *and *ysgA *genes of the *E. coli *K12 genome. Among different extraintestinal pathogenic *E. coli *of the B2 phylogenetic group, 61.7% of pathogenic *Escherichia coli*, 80.6% of human uropathogenic *E.coli *and 94.1% of human neonatal meningitis-causing *E. coli*, respectively, harbor a complete copy of this island; whereas, only a few avian fecal *E. coli *strains contained the complete island. Functional analysis showed that Tkt1 confers very little transketolase activity but is involved in peptide nitrogen metabolism.

**Conclusion:**

These results suggest *tkt1 *and its corresponding genomic island are frequently associated with avian and human ExPEC and are involved in bipeptide metabolism.

## Background

Extraintestinal pathogenic *Escherichia coli *(ExPEC) including uropathogenic *E. coli *(UPEC), neonatal meningitis *E. coli *(NMEC), and avian pathogenic *E. coli *(APEC), cause infection in humans and/or animals [1]. One of the most common diseases caused by ExPEC in animals is systemic colibacillosis due to APEC that often starts as a respiratory tract infection and progresses to septicemia, which is characterized by fibrinous lesions of the internal organs [2]. A variety of factors have been associated with ExPEC virulence including pilus adhesins, the temperature-sensitive hemagglutinin (Tsh), serum resistance traits (e.g., *iss *and *traT*), iron acquisition systems (e.g., aerobactin, salmochelin and yersiniabactin), and vacuolating autotransporter toxin (Vat) [2,3]. Chromosomally located virulence genes occur widely among all ExPEC subpathotypes [4,5], but plasmid-linked virulence genes are more common in APEC and NMEC subpathotypes than they are in UPEC [5]. It is also well known that ExPEC strains often contain multiple pathogenicity islands (PAIs), which are horizontally acquired genomic regions of 20 to 200 kb. PAIs are present in pathogenic bacteria but absent from *E. coli *K12, and carry genes encoding one or more virulence factors.

Since they are horizontally acquired, they differ from the rest of the genome in G+C content and codon usage [6]. The first PAI identified on the APEC chromosome was the VAT-PAI, which contains the vacuolating autotransporter gene, *vat*, a contributor to APEC virulence. *vat *has been reported to be present in about half of the APEC, UPEC, and NMEC strains [7]. A *selC*-associated genomic island of APEC strain BEN2908 was subsequently described. This island is prevalent in ExPEC strains and is involved in carbohydrate uptake and virulence [8]. Two PAIs were characterized in APEC O1. One is the PAI localized in the large plasmid pAPEC-O1-ColBM [9,10], and the other is PAI I_APEC-O1_, harboring *ireA*, the *pap *operon and the invasion locus *tia *[11]. The PAI I_APEC-O1_-related genes occurred not only in strains belonging to the APEC subpathotype (17.9%) but also in UPEC (10.7%) and NMEC (28.0%).

In a previous study we used signature-tagged transposon mutagenesis (STM) to identify 28 virulence-associated genes in APEC [12]. One of the genes identified, *tkt1*, encodes a transketolase-like protein whose amino acid sequence shares 68% identity to TktA of a *Vibrio cholerae *strain [13]. However, it does not show any similarity with the *tktA *gene of *E. coli *MG1655 at the nucleotide level. Recent completion of the first APEC genomic sequence (APEC O1) showed that *tkt1 *is localized on an 'as-yet' uncharacterized genomic island [14]. Here, we sought to better understand the prevalence and function of *tkt1 *and its associated genomic island in APEC pathogenicity.

## Methods

### Bacterial strains, plasmids and growth conditions

All bacterial strains and plasmids used in this study are listed in Table 1. APEC O1, an *E. coli *O1:K1:H7 strain that shares strong similarities with sequenced human ExPEC genomes [14], was used to construct the mutants and as a positive control in virulence and other functional assays. A *tktA *mutant, BJ502 of an *E. coli *K12 strain, was used as the control strain in the functional analysis of APEC O1 mutants [15], and *E. coli *DH5α was employed as a negative control in the virulence assays. A well-characterized collection of APEC, fecal *E. coli *isolated from the feces of healthy birds (avian fecal *E. coli*), human UPEC, and human NMEC were used for gene prevalence studies. Strains were grouped phylogenetically using multiplex PCR [16]. Cells were routinely grown at 37°C in Luria Bertani broth (LB) supplemented with an appropriate antibiotic: kanamycin (Km; 50 mg ml^-1^), chloramphenicol (Cm; 25 mg ml^-1^), or ampicillin (Amp; 100 mg ml^-1^), unless otherwise specified.

**Table 1:** Bacterial strains and plasmids used in this study

Strain	Description	Reference
APEC O1	O1:K1:H7; *fyuA, sitA, chuA, irp2, iroN, ireA, tsh, iucD, fimC, iss, ompA, vat, traT*; contains four plasmids, including pAPEC-O1-ColBM	[14]
BJ502	*E. coli *K12, Δ*tktA *	[15]
DH5α	*E. coli *K12	
APEC O1-M*_tkt1_*	APEC O1 derivative, Δ*tkt1*	this study
APEC O1-M*_tktA_*	APEC O1 derivative, Δ*tktA*	this study
S17λpir	*recA thi pro hsdR*^- ^*M^+ ^*RP4::2-Tc::Mu::Km Tn*7 *lysogenized with *λpir *phage	[12]
S17pGP *tkt1*	S17λpir with plasmid pGP704 *tkt1*	this study
APEC O1-C*_tkt1_*	APEC O1 M*_tkt1 _*with plasmid pGP *tkt1 *inserted into bacterial chromosome	this study
APEC O1-P1	APEC O1 M*_tkt1 _*with plasmid pBAD *tkt1*	this study
BJ502-P1	BJ502 with plasmid pBAD24	this study
BJ502-P2	BJ502 with plasmid pBAD *tkt1*	this study
BJ502-P3	BJ502 with plasmid pBAD *tktA*	this study
APEC collection	452 APEC strains isolated from lesions of birds clinically diagnosed with colibacillosis	[17]
Avian fecal *E. coli*	106 avian fecal *E. coli *strains were isolated from the feces of apparently healthy birds	[17]
UPEC collection	200 uropathogenic *E. coli *strains from from MeritCare Medical Center in Fargo, North Dakota	[18]
NMEC collection	91 human neonatal meningitis-causing *E. coli *strains from the cerebrospinal fluid of newborns in the Netherlands, isolated from 1989 through 1997 and from Dr. K. S. Kim at John Hopkins.	[19]
**Plasmids**		
pGP704	Ap^r^, suicide plasmid	[20]
pBAD24	Ap^r^, expression plasmid with arabinose-inducible promoter	[21]
pKD46	Ap^r^; expresses λ red recombinase	[22]
pKD3	*cat *gene, template plasmid	[22]
pGP *tkt1*	pGP704 derivative harboring *tkt1 *gene	this study
pBAD *tkt1*	pBAD24 derivative, *tkt1 *gene under the control of P_BAD_	this study
pBAD *tktA*	pBAD24 derivative, *tktA *gene under the control of P_BAD_	this study

### PCR and multiplex PCR

DNA templates were prepared by the rapid boiling-lysis method. Primer pairs used were tkt1- F 5'- cttacggcggtactttcctg-3'and tkt 1-R 5'-gtacgccgcatcctgattat-3'; genomic island left junction primer pair piaL-F 5'-cgacatcatggattcgattg-3'and piaL-R 5'-ggatggtgctggatcgtact-3'; and genomic island right junction primer pair piaR-F 5'-gcgccactcttcttctgttc-3' and piaR-R 5'-tcagctaattgctcggcttt-3' PCR was accomplished under the following reaction conditions: 4 mM magnesium chloride, 0.25 mM deoxynucleotide triphosphates 0.3 uM each primer, and 1 Unit *Taq *DNA polymerase. Reactions were performed in a Mastercycler EP machine (Eppendorf, Germany) using the following cycling parameters: 94°C for 4 min; 30 cycles of 94°C for 30 sec, 55°C for 30 sec, 72°C for 2 min; and a final cycle of 72°C for 10 min.

### Construction of plasmids, mutants and complemented strains

Enzymes used for generation of constructs were purchased from New England Biolabs. The pBAD expression system (Invitrogen) was used for cloning and arabinose-inducible expression of *tkt1 *and *tktA*. The coding sequence of *tkt1 *was amplified by PCR using genomic DNA of APEC O1 as the template. The Advantage™ 2 PCR kit (Clontech, Mountain View, CA) was used in these experiments according to the manufacturer's directions. The primers used for *tkt1 *gene were the tkt1_E_-F primer 5'-agctccatggattcacaattactggctaacg-3', which introduces an *Ncol *site (underlined bases) and the tkt1_E_-R primer 5'- gcattctagagtcatcctttcaccccttgtgcag-3' which introduces an *Xba*I site (underlined bases). The primers used for *tktA *were tktA_E_-F 5'-agctccatggcctcacgtaaagagcttgcc-3'and tktA_E_-R 5' gcattctagattgcggcccttctcacaaagcat-3' The complete *tkt1 *gene and *tktA *were cloned into the expression vector pBAD24 using the created *Nco*I and *Xba*I sites [21] to obtain pBAD *tkt1 *and pBAD *tktA*, respectively (Table 1). The APEC O1 mutant strain APEC O1 M*_tkt1 _*with plasmid pBAD *tkt1 *was designated as APEC O1-P1, and the *E. coli *K12 mutant strains BJ502 harboring the empty pBAD24, pBAD *tkt1 *and pBAD *tktA *plasmids were designated as BJ502 p1, BJ502 p2 and BJ502 p3, respectively.

Deletion of *tkt1 *was achieved using the method of Datsenko and Wanner [22]. The Cm resistance cassette in pKD3, flanked by 5' and 3' sequences of *tkt1*, was amplified from genomic DNA of strain APEC O1 using primers tkt1_M_-F (5'-ttagcgggctggtttcagcccgccagacagagagagctgaagtgtgtaggctggagctgcttcga-3') and tkt1_M_-R (5'-tcaaggggtaaaaggtcatcctttcaccccttgtgcaggtcatatgaatatcctccttag-3') and was introduced into APEC O1 by homologous recombination using λ Red recombinase. Successful Δ*tkt1*::Cm mutation was confirmed by PCR, using primers flanking the *tkt1 *region. The Δ*tkt1*::Cm derivative of APEC O1 was designated APEC O1 M*_tkt1_*. The mutant strain APEC O1 M*_tktA _*(Table 1), a Δ*tktA*::Cm derivative of APEC O1, was generated using primer pair tktA_M_-F 5'-aagggccgcatttgcggcccttctcacaaagcatcttaccgagtgtaggctggagctgcttcga-3' and tktA_M_-R 5'-cgttaagggcgtgcccttcatcatccgatctggagtcaaacatatgaatatcctccttag-3'. The Δ*tkt1 *mutant strain APEC O1 M*_tkt1_*, was complemented by single-copy integration of the plasmid pGP*tkt1*. The *tkt1 *operon, including the 300-bp upstream DNA sequence, was amplified by PCR using primers tkt1_C_**-**F 5'**-**tgacagatctgggctatgcagcgatttactac-3' and tkt1_C_-R 5'-cagttctagatgtgcaggtttagctgttcagt-3'. Plasmid pGP*tkt1 *was constructed by cloning this *Bgl*II-*Xba*I (underlined bases) fragment into the same sites of suicide vector pGP704 [10,20]. PGP*tkt1 *was conjugated from strain S17- pGP*tkt1 *to strain APEC O1 M*_tkt1_*. A strain that was resistant to Amp and found to contain a full-length copy of the *tkt1 *gene, as confirmed by PCR, was designated APEC O1 C*_tkt1 _*(Table 1).

### Phenotype microarray

The Phenotype Microarray (PM) assay was performed essentially according to published methods using Biolog PM plates (Biolog Inc., CA). APEC O1 and APEC O1Δ*tkt1 *were grown overnight at 37°C in BUG_B agar (Biolog Inc., CA). Cells were washed with IF-0 GN Base inoculating fluid (Biolog Inc., CA), and then resuspended in IF-10 GN Base inoculating fluid (Biolog Inc., CA) at a density corresponding to 85% transmittance (approximately 0.185 OD600 nm). The suspensions were then inoculated into microplate PM1-8 for the metabolism test (Biolog Inc., CA) at a volume of 100 μl per well. Cell growth was monitored by measuring the respiration-dependent color change of tetrazolium violet in each well. The PM assay was performed twice.

## Results

### *tkt1 is *strongly associated with APEC and ExPEC of the B2 phylogenetic group

**Figure 1:**
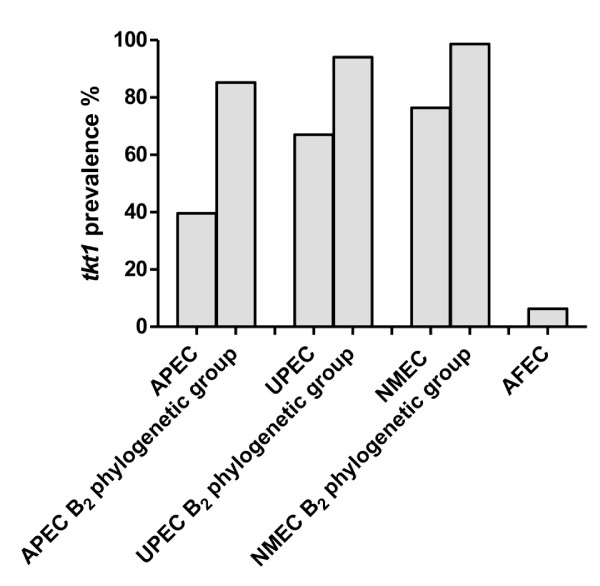
**Prevalence of *tkt1 *in ExPEC strains**.

*tkt1 *was initially identified as an APEC-specific gene by genomic subtraction [23]. Here, we examined its prevalence in a collection of APE and avian fecal *E. coli*. A pair of primers designated tkt1F and tkt1R (Table 1) were used to test 96 APEC and 48 avian fecal *E. coli *strains by PCR. Thirty-eight strains from the APEC group (39.6%) were positive for *tkt1*; while only three strains from avian fecal *E. coli *group were positive (6.25%). Thus, *tkt1 *is significantly more likely to be present in pathogenic strains (*P *< 0.001). Interestingly, 12 out of 14 (85.7%) APEC strains from phylogenetic group B2 were *tkt1 *positive; while the prevalence of *tkt1 *in APEC strains from any other phylogenetic group was much lower (group A, 16.1%; group B1, 12.5% and group D, 47.4%). Since only 14 strains of phylogenetic group B2 were used, a number inadequate for statistical analysis, an additional 47 APEC strains of phylogenetic B2 group were selected from our collection and examined. A total of 52 out of 61 APEC (85.2%) from phylogenetic group B2 was found to be positive for *tkt1 *(Figure 1), demonstrating that *tkt1 *is significantly (*P *< 0.01) associated with APEC strains belonging to phylogenetic group B2.

Several recent studies have shown that most human ExPEC strains belong to the B2 phylogenetic group [4,24], and analysis of the genomic sequences of UPEC strains CFT073 and 536 revealed that they contained *tkt1*. Such results suggest that *tkt1 *might also be prevalent among human ExPEC. To verify this hypothesis, 94 UPEC strains and 89 NMEC strains were examined by PCR for the presence of *tkt1*. As expected, 67% of UPEC and 76.4% of NMEC strains were positive for *tkt1 *gene. As was the case with APEC, the majority of UPEC (94%) and NMEC (98.6%) belonging to phylogenetic group B2 were positive for *tkt1*. Therefore, *tkt1 *gene has been significantly associated with ExPEC strains from human and avian hosts, especially with strains of phylogenetic group B2.

### *tkt1 *is located on a novel genomic island prevalent in ExPEC of the B2 phylogenetic group

**Figure 2:**
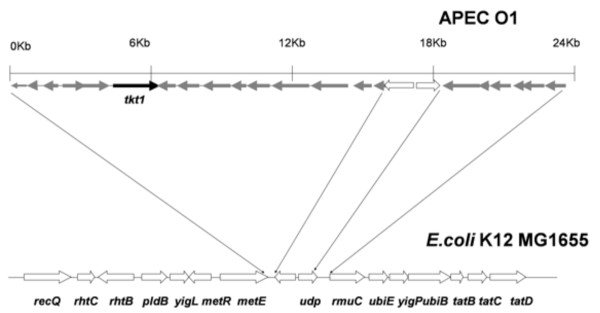
**Genetic organization of the 16 Kb *tkt1 *genomic island and its flanking regions within the APEC O1 genome, drawn to scale**. The ORFs present in this genomic island are listed in the Table 2. There is an islet containing 6 ORFs between the *udp *and *rmuC *genes.

Study of the genomic sequences of several ExPEC showed that *tkt1 *is located in a 16-kbp genomic island inserted between *metE *and *ysgA *[14,25,26]. The overall G+C content of this island is 48.57%, whereas the average G+C content of the *E. coli *K-12 genome is 50.8%. This discrepancy in G+C content suggests that this particular stretch of DNA does not belong to the *E. coli *backbone and is foreign. The entire genomic island contains 15 ORFs, including *tkt1*, with the function of most of them 'as yet' unknown. Products encoded by certain ORFs have been assigned hypothetical functions, including a putative permease, putative glucose-specific IIBC component of a PTS system, carbonate kinase-like protein, and putative transcriptional regulators. Besides this genomic island, there is another small genomic islet of about 5 Kb located between the *udp *and *rmuC *genes. This small islet contains 6 ORFs with unknown functions (Figure 2).

**Figure 3:**
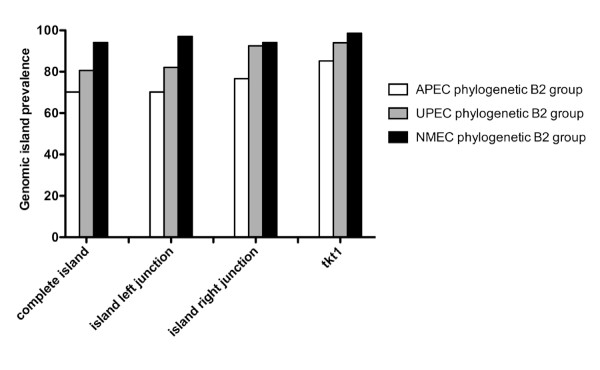
**The prevalence of *tkt1 *genomic island in phylogenetic group B2 of ExPEC strains**.

A multiplex PCR panel was developed to determine the presence of the *tkt1*-containing genomic island in ExPEC of the B2 phylogenetic group. Three pairs of primers were designed to amplify the left and right junctions, as well as the *tkt1 *gene in 61 APEC, 67 UPEC and 68 NMEC belonging to phylogenetic group B2. The results suggest that 70.2% of APEC, 80.6% of UPEC and 94.1% of NMEC strains from B2 phylogenetic group carry a complete copy of this genomic island (Figure 3). Thus, these data demonstrate that this genomic island is significantly associated with ExPEC strains belonging to the B2 phylogenetic group.

### Tkt1 could not complement TktA in *E. coli *K12

**Figure 4:**
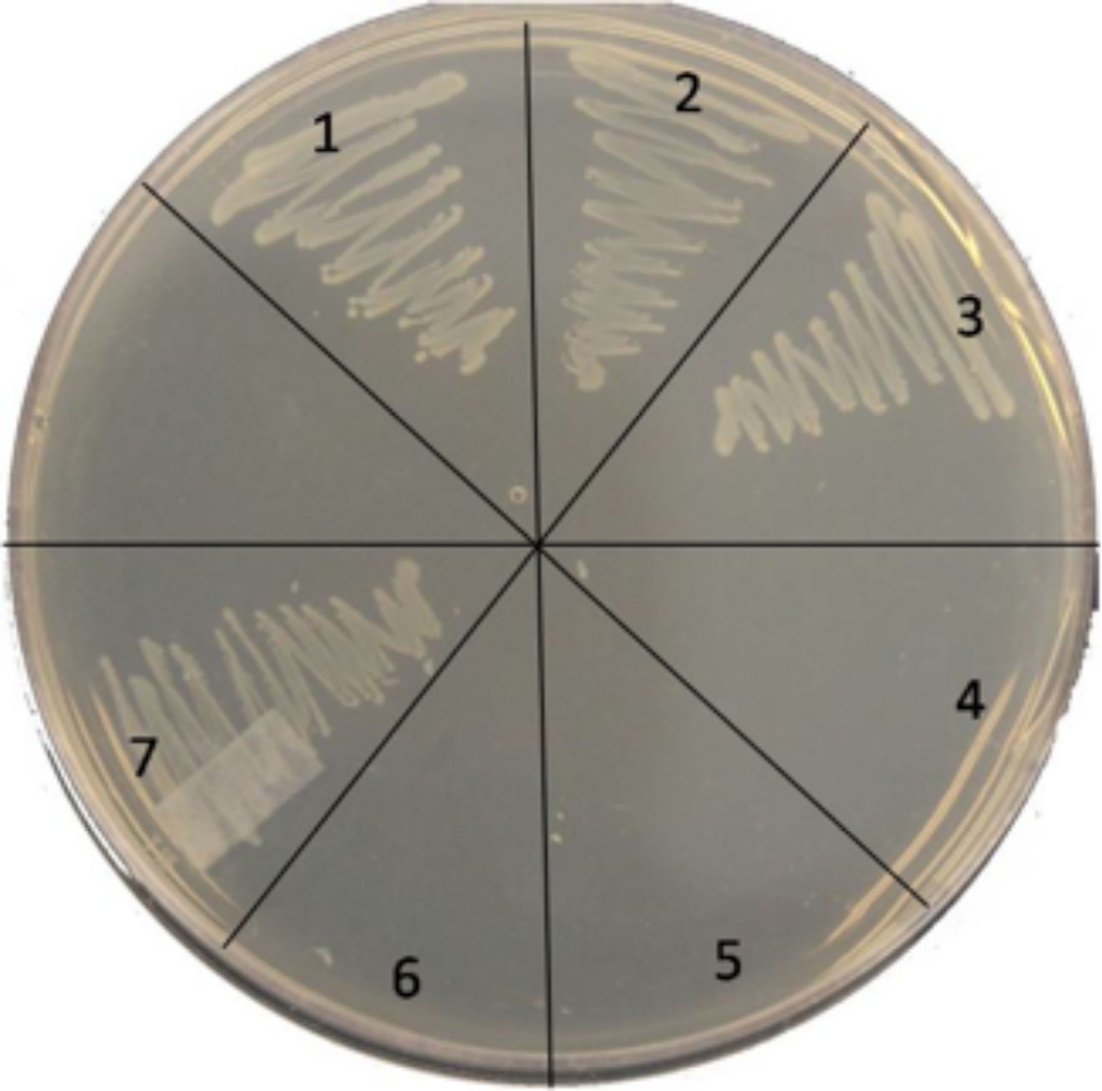
**Tkt1 could not complement TktA in *E. coli *K12. 1, APEC O1; 2, APEC O1 M*_tkt1_*; 3, APEC O1 M*_tktA_*; 4, BJ502; 5, BJ502-P1; 6, BJ502-P2 and 7 BJ502-P3**.

Recently, genome sequencing of APEC O1 revealed that *tkt1 *gene encodes a transketolase-like protein whose amino acid sequence shares 68% identity to TktA of a *V. cholerae *strain [13], although *tkt1 *does not show any similarity to *tktA *of *E. coli *MG1655 at the nucleotide level. To explore the function of Tkt1, mutants with single deletions of *tkt1 *and *tktA *were constructed in the APEC O1 strain using the method of Datsenko and Wanner [22], and their growth was compared to each other and the wild type in M9 plates with L-arabinose as the sole carbon source. The results showed that both mutants of APEC O1 were able to grow in M9 with the *tktA *mutant growing slightly slower than the *tkt1 *mutant. However, the control strain *E. coli *K12 BJ502, which has a mutation in the *tktA*, failed to grow in M9 plates with L-arabinose (Figure 4) [15]. These results suggested that, APEC O1 has another gene that is capable of complementing the *tktA *mutation. To ascertain if it were *tkt1*, a Tkt1 over-expression plasmid pBAD *tkt1 *and a TktA over-expression plasmid pBAD *tktA*, in which the *tkt1 *or the *tktA *gene is under the control of the P_BAD _promoter, were constructed. Induction of TktA expression could recover growth of BJ502-P3 on M9 plates with L-arabinose as the sole carbon source, while Tkt1 expression could not recover growth of BJ502-P2 (Figure 4). These results suggested that Tkt1 has very little transketolase activity, if any.

### Tkt1 is involved in peptide nitrogen metabolism

Transketolase TktA is involved in carbon metabolism, and Tkt1 shows a high similarity (68%) to transketolase TktA. To determine if this transketolase-like protein is involved in metabolism, we performed the PM assay under a total of 760 culture conditions (carbon sources, nitrogen sources, phosphorus and sulfur sources, nutrient supplements, and peptide nitrogen sources). Growth of wild-type APEC O1 and its *tkt1 *isogenic mutant was measured using the PM assay system. The time course of cell growth was monitored by measuring the cell density-dependent increase in respiration. No difference between the *tkt1 *mutant and its wild type in the utilization of carbon sources was detected nor were differences in the use of nitrogen, phosphorus and sulfur sources or nutrient supplements observed. Interestingly, the *tkt1 *mutant showed defects in the use of Pro-Ala or Phe-Ala as a peptide nitrogen source. These defective phenotypes were reproducible, and induction of Tkt1 expression in APEC O1-P1 resulted in the use of both peptides as nitrogen sources reverting the lost phenotype. Complementation assay was also done by using Biolog plates and 0.2% arabinose was added to induce expression of Tkt1.

## Discussion

Human and avian ExPEC are both important pathogens that cause widely prevalent and/or highly significant extraintestinal diseases. The gene *tkt1*, encoding a transketolase-like protein and sharing 68% amino acid identity with TktA of a *V. cholerae *strain [13], was firstly identified as a virulence-associated gene from APEC strains by genomic subtractive hybridization [23]. This gene was also thought to be involved in APEC virulence from the results of a previous STM study [12]. Unlike *tktA *or *tktB*, which are unequivocally present in both avian fecal *E. coli *and APEC, *tkt1 *was predominantly present among APEC (39.6%) but absent from most of the intestinal *E. coli *(6.25%) examined [27], suggesting that this gene may play a significant role in the pathogenesis of avian colibacillosis. APEC and human ExPEC have recently been shown to possess remarkable similarities in their disease-causing abilities, serogroups associated with disease, and virulence genotypes and traits, such as the presence of adhesins, iron acquisition systems, toxins, protectins and invasins that enable them to grow and cause disease in the extraintestinal host environments [5,28]. The inference of a close genetic relationship between APEC and human ExPEC strains was further substantiated by the distribution of *tkt1*. About 67% of UPEC and 76.4% of NMEC strains examined in this study harbor *tkt1*. Like many other virulence genes of ExPEC, *tkt1 *is also phylogenetically distributed. Of the ExPEC belonging to B2 phylogenetic group, 85.2% APEC, 94.0% of UPEC and 98.6% of NMEC were positive for *tkt1*. *E. coli *from phylogenetic group B2 have already been experimentally and epidemiologically associated with extraintestinal infections [29,30]. These results also suggest that *tkt1 *may play a role in the pathogenesis of human ExPEC as well as APEC.

Genomic sequencing of APEC O1 revealed more than 40 genomic islands; several of them are theoretically involved in virulence [9]. Common features of most, if not all PAIs, include that they encode one or more virulence factors; range in size from 10 to 200 kb; and are likely introduced into the genome via horizontal transfer, resulting in G-C ratios and codon usage that may deviate from the organism's typical pattern. Often PAIs are flanked by small direct repeats and are associated with the 3' end of tRNA genes. PAIS may be phage-derived, but some are thought to originate from plasmids. They may contain mobility elements, such as integrons, transposons, and insertion sequences, and if they move, are likely carried on plasmids, conjugative transposons, or phages, whose loss may spontaneously convert a virulent into an avirulent organism [6]. Similarly, the genomic island encoding *tkt1 *is 16 Kb in size and present in the APEC O1 genome but absent from the sequenced genome of the *E*. *coli *K12 strain MG1655. Moreover, the overall G+C content of this island is 48.57%, whereas the average G+C content of the *E. coli *K-12 genome is 50.8%. This discrepancy in G+C content further suggests that this particular stretch of DNA does not belong to the *E*. *coli *K12 backbone and is foreign-derived. Also, the genomic island encoding *tkt1 *is localized in close proximity to tRNA genes. Unlike classical PAIs, no flanking direct repeats or mobility elements such as integrases or transposases were found in this genomic island.

However, such mobility elements may have been lost during the evolutionary process. Horizontal transfer of genes by genomic islands or PAIs is a common phenomenon in extracellular bacterial pathogens. The acquisition of genes in this way allows bacteria to adapt to a new or changing environment thus contributing to the fitness and/or virulence of the recipient organism.

PAIs have been described in several well-known ExPEC strains, including *E. coli *strains 536, CFT073, J96, UTI189, RS218 and APEC O1. Indeed, comparative analysis of the APEC O1 genome and other ExPEC genomes revealed that APEC and human ExPEC share more than 28 pathogenicity (genomic) islands [9,25,26,31]. Among them, the genomic island encoding *tkt1 *was notable in that it was found among all sequenced ExPEC genomes. The multiplex PCR results of this study further demonstrated that a complete copy of this genomic island is significantly associated with both avian and human ExPEC strains of phylogenetic group B2. These observations suggest that the *tkt1 *genomic island may contribute to the virulence/fitness of both avian and human ExPEC.

Though Tkt1 shares 68% amino acid identity with TktA of a *V. cholerae *strain [13], it does not show any homology at the nucleotide level with *tktA *of *E. coli *MG1655. In *E. coli *K12, *tktA *encodes the transketolase A, which is responsible for the major enzymatic activity of transketolase in *E. coli*. Transketolase is a link between glycolysis and the pentose phosphate pathway and is involved in the catabolism of pentose sugars, formation of D-ribose 5-phosphate, and provision of D-erythrose 4-phosphate which is a precursor of aromatic amino acids, aromatic vitamins and pyridoxine [32]. A previous study showed that the *E. coli *K12 mutant BJ502 that carries a mutation in *tktA *was unable to use L-arabinose or D-Xylose as the sole carbon source and required aromatic acids for growth on a minimal medium. The functional analysis in this study demonstrated that over-expression of Tkt1 in *E. coli *K12 mutant strain BJ502 could not recover its growth in M9 medium with L-arabinose as the sole carbon source; while over-expression of TktA could. These results suggest that *tkt1 *could not complement the *tktA *mutation in *E. coli *K12 and Tkt1 confers very little transketolase activity, if any.

Most studies of bacterial pathogenesis have focused on classical virulence factors such as toxins, adhesins, iron uptake systems and factors that confer resistance to innate and adaptive immune mechanisms. However, the role of metabolism in virulence and fitness of bacterial pathogens is becoming better appreciated. Recently, a *selC*-associated genomic island of APEC strain BEN2908 was found to be involved in carbohydrate uptake and virulence [8]. Also in the same APEC strain, a carbohydrate metabolic operon (*frz*) that is highly associated with ExPEC promotes fitness under stressful conditions and invasion of eukaryotic cells [33]. Our STM results showed that one *tkt1 *STM-mutant was out-competed by the wild type from two to a thousand fold in lung, heart, liver, kidney and spleen of 5-week-old chickens. The functional analysis using phenotype microarray revealed that a *tkt1 *mutant has defects in use of Pro-Ala or Phe-Ala as a nitrogen source. These results strongly suggest that *tkt1 *is involved in bipeptide metabolism and contributes to fitness and virulence of APEC. Interestingly, dipeptide transport proteins, DppA and OppA, were identified to be up-regulated when UPEC strain CFT073 was cultured in human urine compared to CFT073 cultured in LB depleted with iron [34]. The greatest challenges confronted by bacterial pathogens are environmental changes, including the rapid changes they encounter in nutrient availability [35]. In the course of evolution, pathogenic organisms have developed several mechanisms to sense and utilize available nutrient sources associated with particular niches or to favor the most efficiently metabolizable nutrient sources when exposed to a range of choices [36]. Thus, genes involved in metabolism, which are required for bacterial growth in specific infection sites, contribute to fitness and virulence. On the other hand, the efficiency of metabolism of a nutrient source or the presence of a specific nutrient source might serve as a signal to switch 'on' or 'off' specific virulence genes in particular infection niches [36].

## Conclusions

A previously identified virulence-associated gene *tkt1 *was further characterized in this study. The results demonstrated that this gene is strongly associated with ExPEC strains of phylogenetic group B2 from human and avian origin and is localized in a genomic island. Function analyses showed that Tkt1 has very little transketolase activity and seems to be involved in peptide nitrogen metabolism.

## Authors' contributions

The project was designed by GL, LN, LW. Experiments were performed by GL, SK,KT, YW, CL under supervision of GL and LN. The paper was co-drafted by LG and LN. All authors approved the final version of the manuscript.

**Table 2:** ORFs present within the *tkt1 *genomic island

**ORF No**.	ORF name Location of ORF Function		G+C content
APECO1_2646		4312693..4312950 hypothetical protein	NC_008563
APECO1_2645		4312947..4313438 hypothetical protein	NC_008563
APECO1_2644		4313787..4314080 hypothetical protein	NC_008563
APECO1_2643		4314532..4315122 putative sugar isomerase	NC_008563
APECO1_2642		4315164..4316672 PTS system, glucose-specific IIBC component	NC_008563
APECO1_2641	*tkt1*	4316723..4318720 Putative transketolase	NC_008563
APECO1_2640		4318750..4319595 putative transcriptional regulatory	NC_008563
APECO1_2639		4319796..4320701 putative transcriptional regulatory	NC_008563
APECO1_2638		4320779..4322002 putative permease	NC_008563
APECO1_2637		4322028..4322417 hypothetical protein	NC_008563
APECO1_2636		4322434..4323390 catalyzes the reversible synthesis of carbamate	NC_008563
		and ATP from carbamoyl phosphate and ADP	
APECO1_2635	*yahG*	4323383..4324858 hypothetical protein	NC_008563
APECO1_2634	*yahF*	4324804..4326363 hypothetical protein	NC_008563
APECO1_2633	*yahE*	4326458..4327318 hypothetical protein	NC_008563
APECO1_2632		4327324..4327992 putative isochorismatase hydrolase	NC_008563
